# Clinical Predictors of Monkeypox Diagnosis: A Case-Control Study in a Nonendemic Region during the 2022 Outbreak

**DOI:** 10.3390/microorganisms11092287

**Published:** 2023-09-11

**Authors:** Alberto Kousuke De la Herrán-Arita, Cuitláhuac González-Galindo, Gerardo Kenny Inzunza-Leyva, Marco Antonio Valdez-Flores, Claudia Desiree Norzagaray-Valenzuela, Alejandro Camacho-Zamora, José Candelario Batiz-Beltrán, Francisco Javier Urrea-Ramírez, Alejandra Romero-Utrilla, Carla Angulo-Rojo, Alma Marlene Guadrón-Llanos, Verónica Judith Picos-Cárdenas, Josué Camberos-Barraza, Ángel Radamés Rábago-Monzón, Juan Fidel Osuna-Ramos

**Affiliations:** 1 Facultad de Medicina, Universidad Autónoma de Sinaloa, Culiacán Rosales 80019, Sinaloa, Mexico; alberto.kousuke@uas.edu.mx (A.K.D.l.H.-A.); marco.valdez@uas.edu.mx (M.A.V.-F.); alejandrocamacho@uas.edu.mx (A.C.-Z.); josebatiz@issste.gob.mx (J.C.B.-B.); francisco.urrea@issste.gob.mx (F.J.U.-R.); carla.angulo@uas.edu.mx (C.A.-R.); almaguadron@uas.edu.mx (A.M.G.-L.); veronicapicos@uas.edu.mx (V.J.P.-C.); rabagoradames.fm@uas.edu.mx (Á.R.R.-M.); 2 Secretaría de Salud de Sinaloa, Culiacán Rosales 80020, Sinaloa, Mexico; cuitlahuac.gonzalez@sinaloa.gob.mx; 3 Dirección de Prevención y Promoción de la Salud, Secretaría de Salud de Sinaloa, Culiacán Rosales 80020, Sinaloa, Mexico; gerardo.inzunza@saludsinaloa.gob.mx; 4 Unidad de Investigación, Facultad de Biología, Universidad Autónoma de Sinaloa, Culiacán Rosales 80040, Sinaloa, Mexico; claudia.norzagaray@uas.edu.mx; 5 Hospital Regional Dr. Manuel Cárdenas de la Vega, ISSSTE, Culiacán Rosales 80230, Sinaloa, Mexico; 6 Departamento de Anatomía Patológica, Instituto Mexicano del Seguro Social, Culiacán Rosales 80230, Sinaloa, Mexico; 7 Maestría en Ciencias en Biomedicina Molecular, Facultad de Medicina, Universidad Autónoma de Sinaloa, Culiacán Rosales 80019, Sinaloa, Mexico; 8 Doctorado en Ciencias en Biomedicina Molecular, Facultad de Medicina, Universidad Autónoma de Sinaloa, Culiacán Rosales 80019, Sinaloa, Mexico

**Keywords:** monkeypox (Mpox), case–control study, clinical diagnosis, clinical predictors, logistic regression, ROC curves, nonendemic region, outbreak, Mpox epidemiology

## Abstract

Monkeypox (Mpox) is an emerging zoonotic disease with the potential for severe complications. Early identification and diagnosis are essential to prompt treatment, control its spread, and reduce the risk of human-to-human transmission. This study aimed to develop a clinical diagnostic tool and describe the clinical and sociodemographic features of 19 PCR-confirmed Mpox cases during an outbreak in a nonendemic region of northwestern Mexico. The median age of patients was 35 years, and most were male. Mpox-positive patients commonly reported symptoms such as fever, lumbago, and asthenia, in addition to experiencing painful ulcers and a high frequency of HIV infection among people living with HIV (PLWH). Two diagnostic models using logistic regression were devised, with the best model exhibiting a prediction accuracy of 0.92 (95% CI: 0.8–1), a sensitivity of 0.86, and a specificity of 0.93. The high predictive values and accuracy of the top-performing model highlight its potential to significantly improve early Mpox diagnosis and treatment in clinical settings, aiding in the control of future outbreaks.

## 1. Introduction

Monkeypox is an emerging disease caused by the Mpox virus, a close relative of the smallpox and cowpox viruses [1]. Clinical symptoms and systemic manifestations include headache, myalgia, backache, and lymphadenopathy, ranging from moderate to severe, appearing days to weeks post-exposure [2,3]. Concurrently, self-resolving skin lesions may emerge, initiating facially before spreading to other body parts, presenting as a vesicular and pustular rash [4]. Sometimes, these lesions require weeks to heal, leaving behind scarring and pigmentation changes [2,3,5,6]. Mortality rates vary from 1% to 10%, significantly lower than smallpox [7,8].

Risk factors for acquiring the Mpox virus include direct contact with an infected individual and specific sexual practices that facilitate direct person-to-person transmission, thereby increasing the likelihood of infection [2,9]. While proximity to or handling infected animals has been traditionally considered a risk factor, it was not the predominant mode of transmission in the most recent outbreak [10]. Early detection and treatment are crucial to mitigate symptom severity, limit disease spread, prevent complications, and improve patient prognosis [11,12]. Diagnosis is primarily clinical, necessitating laboratory testing via antigen-capture ELISA, viral culture, Western blot, and PCR [2]. However, access to these diagnostic procedures may be limited in impoverished and nonendemic regions due to resource constraints, necessitating exploring alternative diagnostic methods [13,14].

The treatment of Mpox disease encompasses symptomatic and supportive care, early detection, and isolation of those infected to prevent the spread of the virus [10]. Since Mpox is an emerging disease and most healthcare providers in nonendemic areas are still unfamiliar, it can be challenging to establish a clinical suspicion of Mpox solely based on symptoms. Therefore, healthcare providers must investigate other causes before diagnosing and treating the disease [1,8]. To provide appropriate diagnosis and treatment, healthcare professionals must comprehensively understand the clinical presentation of Mpox, its symptoms, and the epidemiological exposures associated with the condition [15].

The diagnosis accuracy of Mpox is determined by combining symptomatology or clinical symptoms with laboratory diagnostics [14]. It is also essential to develop a set of recommendations or devices that can assist in identifying the symptoms and avoiding contact with potentially infected persons, thus reducing the risk of transmission and consequences [16]. Early detection of Mpox disease can lessen the effects of signs, limit its spread, and avoid serious complications, as it facilitates the prompt initiation of treatment designed to impede viral replication and dissemination during its developing phase. The administration of antiviral agents such as tecovirimat, cidofovir, and brincidofovir, which have demonstrated efficacy against monkeypox in both laboratory and clinical domains in the immediate aftermath of symptom onset or lesion appearance, holds the promise of alleviating the intensity of Mpox infection [17,18,19].

It can be challenging to predict the severity and rate of progression of Mpox infection; nonetheless, it can considerably reduce the morbidity associated with this disease [12,20]. Accordingly, the main objective of this study was to develop predictive and diagnostic tools for Mpox infections based on clinical features in Mpox-positive patients according to a case–control analysis during the 2022 outbreak in a nonendemic region. In addition, demographic and epidemiological information about the patients was collected to expand our understanding of Mpox.

## 2. Materials and Methods

### 2.1. Study Design and Participants

An observational case–control study was conducted based on epidemiological surveillance data from Sinaloa, Mexico. Data were obtained from the SINAVE (National Epidemiological Surveillance System) system of the Mexican Ministry of Health, which included demographic, epidemiological, and clinical information about the symptoms and skin lesions of Mpox infection from 1 May 2022 to 1 December 2022. Patients with one or more acute skin lesions (of any kind) and at least one of the following symptoms were considered probable Mpox cases: fever, myalgia, headache, lymphadenopathy, fatigue, arthralgia, and lumbago [21]. None of the patients received the Mpox vaccine. Following the guidelines from the Mexican Ministry of Health, no specific vaccine for Mpox is available. The only vaccine related to it is for human smallpox, but it is strictly not recommended for the general public [22].

The attending clinician recorded the onset date of fever, skin lesions, and other general symptoms such as fever, headache, arthralgias, nausea, myalgias, lumbago, vomiting, asthenia, cough, odynophagia, and odynophagia, on the case report form. Patient self-report and physical examination were used to document symptoms and clinical signs. A swab of exudate from skin lesions (vesicles, pustules, and scabs) was collected in suspected cases. It was processed only when a complete clinical history and a parallel collection of skin lesions were provided. RT-PCR was implemented by InDRE (Mexican Epidemiological Diagnosis and Reference Institute) to detect Mpox using the methodology previously described [23]. This criterion identified Mpox-positive patients, whereas PCR-negative samples were classified as controls (Mpox-negative). In addition, standard PCR assays were used for the differential diagnosis of varicella-zoster virus (VZV; human herpesvirus 3) and herpes simplex virus types 1 and 2 (HHV-1 and -2) [24,25,26].

### 2.2. Statistical Analysis

Categorical variables were expressed as numbers and frequencies (%), while continuous variables were expressed as the median and interquartile ranges (IQR). Pearson’s chi-square test was used to compare categorical variables between Mpox-positive and Mpox-negative cases, and the Mann–Whitney U test was used to compare continuous variable differences. Logistic regression was used to calculate coefficients, odds ratios (OR), and 95 percent confidence intervals (CI). The model’s goodness of fit (GOF) was determined using the Hosmer–Lemeshow test. The receiver operating characteristic (ROC) curves and areas under the curves (AUC) were calculated to determine the predictive accuracy. The De Long et al. method estimated the exact binomial 95% CI and standard errors (SEs) for the AUC. R version 4.1.0 and RStudio version 1.3 (R & RStudio, Boston, MA, USA) were utilized for the statistical analysis, and a two-tailed *p*-value of 0.05 was considered statistically significant.

## 3. Results

**Figure 1:**
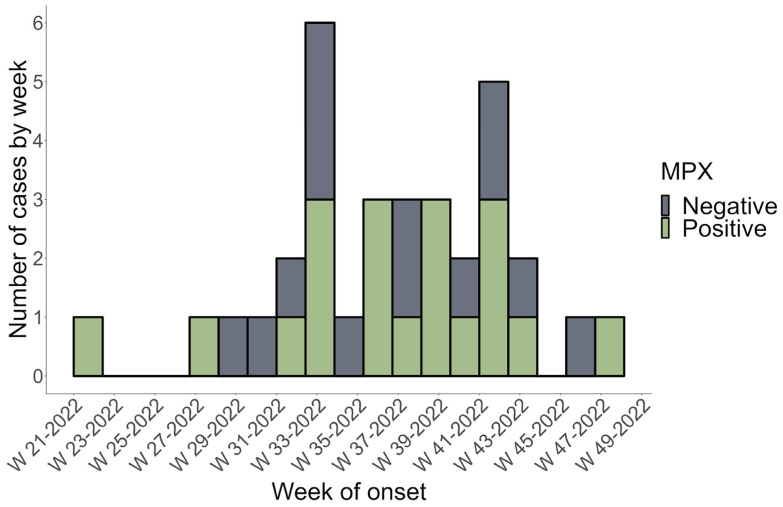
The epidemic curve of Mpox-positive (cases) and Mpox-negative (control) subjects in Sinaloa, Mexico, from 16 May 2022 to 1 December 2022. PCR confirmed a weekly count of positive and negative cases based on the onset date of symptoms (MPX = Mpox).

The current study included 42 subjects suspected of being infected with Mpox. Four patients were excluded due to a lack of data. Therefore, only 38 patients were recruited after PCR testing and Mpox infection confirmation. The 38 patients included in our study were all Mexican nationals. The first positive Mpox case in Sinaloa, Mexico, was recorded on 24 May 2022, as shown in the epidemic curve plotted using data from this study (Figure 1). Figure 1 shows that week 33 had the highest reported cases, with three PCR Mpox-positive and three Mpox-negative patients.

Most Mpox-positive patients were men, with a median age of 35 (Table 1). Mpox-positive patients self-identified as homosexual (28%), bisexual (16%), heterosexual (16%), and male having sexual relations with another man (MSM) (11%). Sexual contact was the most often reported transmission mode (Table 1). According to the RT-PCR results, 19 patients (50%) tested positive for Mpox virus, three patients (7.9%) tested positive for VZV, and the remaining patients (16%) tested harmful for Mpox, making them Mpox-negative subjects (Table 1). Patients who tested positive for VZV had the following characteristics: Patient 1, a 32-year-old man from Culiacán, displayed exanthema on his head, upper limbs, neck, and back, along with a temperature of 39 °C; Patient 2, a 20-year-old from Culiacán, presented with macules, vesicles, pustules, and scabs in multiple regions; Patient 3, a 73-year-old man from Mazatlán, exhibited lesions with similar cephalocaudal distribution. Notably, all tested negative for Mpox via PCR and were considered Mpox-negative subjects. In Table 1, the term ‘unknown’ refers to cases where neither Mpox nor VZV was detected.

All participants in the study received medical care; however, only six (16%) were hospitalized: four tested positive for Mpox, and two tested negative for Mpox (Table 1). Of the individuals studied, only two reported having been in contact with laboratory-confirmed Mpox cases. Both individuals indicated that the exposure occurred within their homes, suggesting person-to-person transmission as the primary mode of spread. The majority of the other participants were unaware of any such contact.

Table 2 and Table 3 summarize the clinical manifestations and skin lesions of Mpox-negative and Mpox-positive individuals, respectively. All Mpox-positive patients reported fever, compared to 74% of Mpox-negative subjects. Other significant differences (*p* < 0.05) were a high prevalence of fever, lumbago, asthenia, and painful ulcers in the Mpox-positive group (Table 2). Mpox-positive patients reported a median of seven symptoms compared to Mpox-negative patients (*p* = 0.015), with most showing more than seven symptoms (*p* = 0.007). Interestingly, a high prevalence of PLWH was also observed among Mpox-positive patients (53%) (*p* = 0.001) (Table 2). Macules (59%), papules (88%), vesicles (89%), pustules (71%), and scratches (76%) were among the skin lesions evaluated in the Mpox-positive patients (Table 3). The chest (76%), face (74%), head (63%), neck (47%), upper limbs (72%), lower limbs (67%), palms (41%), genital area (44%), and abdomen (35%) were the most affected regions in Mpox-positive (Table 3). In our analysis, only the presence of skin lesion papules (*p* = 0.021) and axillary lymphadenopathy (*p* = 0.040) demonstrated statistically significant differences between the Mpox-negative and Mpox-positive groups.

**Figure 2:**
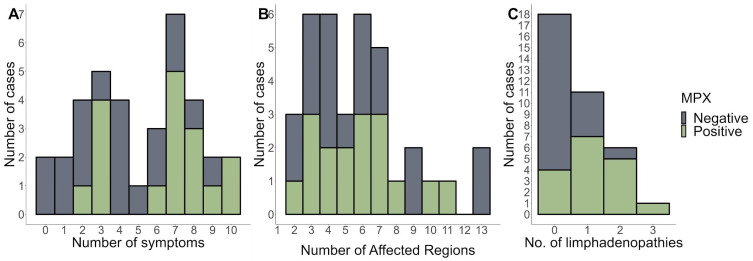
The number of symptoms (**A**), affected regions (**B**), and (**C**) number of lymphadenopathies stratified by MPX-negative and MPX-positive patients.

Figure 2A,B show the distributions of symptoms and several skin-affected regions. Overall, the findings show that MPX-positive patients reported more symptoms and had more affected parts than MPX-negative patients. Furthermore, most patients had lymphadenopathy in multiple regions (Figure 2C). This suggests that MPX-positive patients are more likely to experience symptoms related to their disease and have more body areas affected.

**Figure 3:**
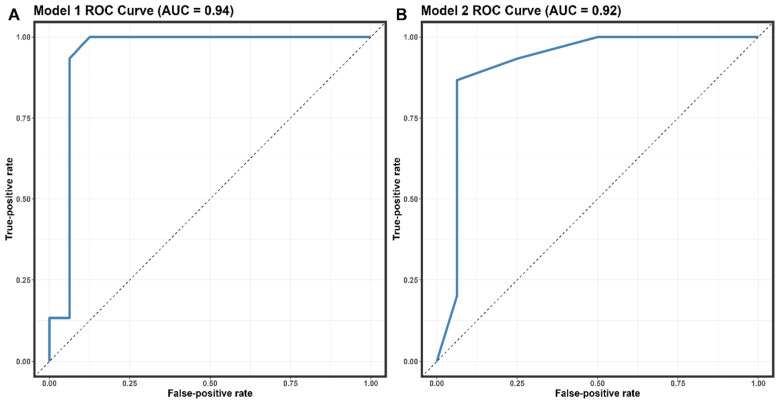
Receiver operating characteristic (ROC) curves the performance of models 1 (**A**) and 2 (**B**)—abbreviation: AUC, area under the receiver operating characteristic curve.

The covariates selected for the logistic regression analysis were the number of clinical symptoms, the number of affected lymph nodes, and the comorbidity of PLWH patients. The multivariate analysis revealed statistically significant differences between MPX-positive and MPX-negative patients. Table 4 details the findings of the univariate study and the two multivariate models. In the univariate model, statistically significant risk variables for an MPX-positive diagnosis were lumbago, asthenia, the presence of papules, the number of symptoms, and the number of lymphadenopathy-affected locations. The second multivariate model found the best fit (*p* = 0.5076). Covariates of the number of symptoms and lymph nodes affected were shown to have high predictive values. We discovered that for >7 symptoms (OR = 10.3; 95% CI, 1.47–119; *p* = 0.03), >1 afflicted area with lymphadenopathy (OR = 8.6; 95% IC 1.23–97.8; *p* = 0.04), and the presence of PLWH comorbidity (OR = 16, 95% CI 1.39–537; *p* = 0.05), there was an increased risk in MPX-positive diagnosis. The predictive performance of Model 2 for distinguishing MPX-positive from MPX-negative was determined to have an AUC of 0.92 (95% CI: 0.8–1, sensitivity = 0.86, specificity = 0.9375) (Figure 3B).

## 4. Discussion

This study describes the clinical and epidemiological features of the first confirmed Mpox cases in Sinaloa, a state in northwest Mexico, during the Mpox 2022 outbreak. The present study’s findings shed more light on the existing data on Mpox in nonendemic regions. Historically, incidences outside Africa were related to foreign travel to endemic areas or imported animals [27,28]. Transmission from person to person is also possible through close contact with an infected individual. Albeit outside endemic regions, epidemiological evidence suggests that interpersonal communication drives the spread of Mpox worldwide [16,29]. The results of the current study suggest a significant increase in MPX cases during week 33 of the outbreak, which corresponds to the highest incidence rate in the country during the study period (1 May 2022 to 1 December 2022) [23]. No seasonal pattern of Mpox outbreaks has been identified in endemic or nonendemic regions; however, peaks of maximum incidence were observed in August–October during previous MPX outbreaks [30,31]. Increased human contact, connectivity, and cessation of smallpox vaccination may have caused the increased human-to-human transmission of Mpox [6,31].

According to the results of this study, most Mpox-positive patients were males, there was a high incidence of PLWH, and high proportions of self-identified homosexuals, bisexuals, heterosexuals, and MSM individuals were found. Mpox is a viral infection that can impact anyone, irrespective of their sexual orientation or gender identity. There is no evidence to suggest that individuals who identify as LGBTQ+ are at an increased risk of contracting Mpox. Therefore, public health efforts should prioritize the LGBTQ+ and MSM communities for prevention and testing while addressing equity, minimizing stigma, and maintaining vigilance for transmission in other populations [6].

In our study, sexually transmitted infections (STIs) were tested based on clinical judgment. While 53% of the patients who tested positive for Mpox were PLWH, specific testing for Ct, Ng, and syphilis was not conducted for all patients. The decision to test for these STIs was based on the clinical presentation and judgment of the attending physician. We acknowledge the importance of comprehensive STI testing, especially given the potential cutaneous involvement of diseases like syphilis, which can present similarly to Mpox [11]. Although there is no direct association between Mpox and HIV, individuals with compromised immune systems, including those with advanced HIV (AIDS), may be particularly susceptible to severe Mpox infection [16,32]. Although simulations based on mathematical modeling indicate that HIV infection exacerbates MPX infection and vice versa, more research is needed to confirm this association and better understand the clinical implications for this population [33]. Furthermore, information on sexual exposure, gender identity, and genital lesions could help explain how the disease spreads. Most Mpox cases observed in the Western world among MSM are due to close skin-to-skin contact; the virus is not conveyed through seminal or vaginal fluid and cannot be considered a sexually transmitted disease [34].

Regarding the clinical manifestations of Mpox infection, we found a high prevalence of fever, headache, arthralgia, lumbago, asthenia, ulcer pain, lymphadenopathies, and skin lesions (Table 2). Although Mpox clinical symptoms have been shown to vary between studied populations [31,35], most of our findings were consistent with the general knowledge of the prodromal stage, which includes symptoms such as fever, muscle pain, muscle/limb pain, lymphadenopathy, skin lesions, and a general feeling of illness. Other previously reported clinical symptoms, such as mucosal lesions, fatigue, night sweats, proctitis, eye involvement, and penile edema, were not found [36]. Together with our findings, these studies highlight the importance of recognizing these clinical signs for early diagnosing and managing Mpox infections.

It is worth noting that the clinical manifestations of monkeypox have evolved and may present differently depending on the population and geographical setting. A recent systematic review by Pourriyahi et al. has characterized monkeypox’s ‘new face,’ particularly focusing on its mucocutaneous presentations [4]. The study describes a distinct pattern in the development of the rash, which can initiate on the face and then spread to other parts of the body, including the trunk and extremities. The rash progresses through macular, papular, vesicular, and pustular stages before eventually crusting over and falling off. This nuanced understanding of the rash’s presentation and progression is crucial for clinicians and epidemiologists, as it provides a more comprehensive view of the disease’s clinical characteristics in the current outbreak.

After an initial prodromal fever, Mpox disease appears with lymphadenopathy and a maculopapular rash with a centrifugal distribution, including lesions on the palms of the hands and soles of the feet. Compared to Mpox-negative patients, Mpox-positive patients reported more symptoms and more affected regions (Figure 2A,B). In this study, three male patients tested positive for varicella-zoster virus (VZV) infection. All patients were negative for Mpox via PCR, ruling out the possibility of a co-infection with VZV and Mpox. It has been reported that varicella is a potential risk factor for monkeypox acquisition in West and Central Africa [24]. In Mexico, where Mpox is not endemic, the likelihood of such co-infections could be negligible. In contrast, in the African context, the severity of the disease was not exacerbated in co-infected individuals compared to those with only monkeypox, indicating that prior VZV infection may have a moderating effect on subsequent monkeypox severity. This highlights the importance of VZV surveillance, especially in regions where monkeypox outbreaks have been reported [24,25,26].

Most patients in our study had lymphadenopathies in multiple regions, consistent with the findings of Nolen et al. (2016), who reported the prevalence of multi-regional lymphadenopathy in Mpox cases [37]. More than seven symptoms indicate a positive Mpox case, whereas fewer than seven symptoms are more indicative of a negative case, as determined by the multiple Mpox criteria. It is important to note that clinical manifestations alone are not sufficient for a definitive diagnosis of Mpox, as they can overlap with other diseases such as chickenpox, measles, bacterial skin infections, scabies, syphilis, and medication-associated allergies. However, lymphadenopathy is a clinical feature that can assist in distinguishing Mpox from chickenpox or smallpox at the prodromal stage [11].

International travel increases the risk of sexually transmitted infections [38]. Matsee et al. conducted a study indicating a considerable increase in demand for international travel in the Southwest Asian region. The increase in international travel poses a significant risk of introducing Mpox, potentially causing new outbreaks [39]. The research underlined a considerable rise of 48.7% over seven days, primarily noted around the beginning of December 2022. In tourist places with significant levels of tourism and frequent meetings and activities, it is necessary to guarantee the effective distribution of critical information about the symptoms, transmission dynamics, and preventative techniques linked with Mpox.

The logistic regression analysis in our study identified the number of clinical symptoms, the number of affected lymph nodes, and the comorbidity of being an AIDS/HIV patient as significant predictors of Mpox infection (Table 4). The predictive performance of Model 2 for distinguishing Mpox-positive from Mpox-negative was determined to have an AUC of 0.92 (95% CI: 0.8–1, sensitivity = 0.86, specificity = 0.9375). These findings suggest that our model can effectively identify patients with MPX infection, which may be particularly useful in areas with limited diagnostic testing capabilities. A recent study conducted in European and non-epidemic populations analyzed the predictive factors of Mpox infection using logistic regression [40]. In this study, similar factors, including the number of clinical symptoms and the presence of PLWH comorbidity, were significant predictors of MPX infection. The predictive performance of their model had an AUC of 0.89 (95% CI: 0.81–0.97), with a sensitivity of 0.82 and a specificity of 0.91 [40]. Although the AUC of our model was slightly higher than that of Moretti et al.’s model, both models demonstrated comparable predictive performance, indicating their potential utility in diagnosing Mpox infection. The differences in the study population, sample size, and methodological approach between our study and Moretti et al.’s study might have contributed to the slight variations in the predictive performance of the models. However, the consistency in the identified predictors across both studies suggests that these factors may significantly affect Mpox infection diagnosis.

Further research, including multicenter studies and external validation analysis, is needed to confirm and refine the predictive factors identified in our study and Moretti et al.’s study. The above elements could contribute to developing more robust, clinically based diagnostic tests for Mpox infection that can be used effectively in real-world settings, as diagnostic techniques in nonendemic settings may be limited. Given the lack of accessibility in developing countries and nonendemic regions, accessing the necessary laboratory equipment and personnel to carry out the required diagnostic tests may be difficult. These clinical-based diagnostic strategies are intended to supplement their application rather than replace it.

While antivirals have been used to treat Mpox, their availability is generally limited to severe cases with complications [41]. Our study did not focus on the treatment aspect but aimed to provide clinicians with a tool for early identification and description of Mpox symptoms and signs. This could be particularly useful in settings where PCR testing is not readily available, although we acknowledge that PCR remains the gold standard for Mpox diagnosis [42].

Despite the valuable insights provided by our study, several limitations should be acknowledged. One major limitation was the small sample size, which may have limited the power of our analyses and not captured some of the clinical characteristics often described in larger cohorts of people diagnosed with Mpox. This restricts the generalizability of our findings and their applicability to international settings. Future studies should aim to include a more extensive and diverse sample to strengthen the generalizability of the results. Second, our study relied on self-reported data for many variables, which may have introduced recall bias or misreporting of information. Objective measures of clinical symptoms and more rigorous data collection methods should be employed in future research to reduce this potential bias. Third, our study was conducted in a single nonendemic region during a specific outbreak, which may limit the applicability of our findings to other geographical areas and periods. Fourth, Mexico’s diagnostic method for Mpox detection involves convalescent serum sampling using PCR methods, but not evaluation of neutralizing antibodies or ELISA. However, these techniques are susceptible to false-negative results due to non-Mpox infections, incorrect selection, or intermittent viral shedding [43,44]. We acknowledge that our testing was limited to one site, which may have hindered our understanding of the extent of Mpox infection. Lastly, the diagnostic tool developed in this study relies on clinical symptoms and epidemiological indicators, which, while useful in resource-limited settings, are not as accurate as laboratory-based diagnostic methods such as PCR testing [42]. Clinical symptoms can overlap with other diseases, and lymphadenopathies can be a distinguishing feature [11]. Therefore, our tool is intended to complement, not replace, PCR-based diagnosis, particularly in settings where laboratory testing is unavailable.

## 5. Conclusions

In conclusion, our study provides valuable insights into Mpox infections’ clinical and epidemiological characteristics in a nonendemic region. We identified key factors, such as the number of clinical symptoms, the number of affected lymph nodes, and the comorbidity of being PLWH, as significant predictors of Mpox infection. These factors could be used to develop a diagnostic tool for identifying Mpox-positive patients in areas with limited diagnostic testing capabilities. Although our findings are subject to certain limitations, they contribute to the growing body of literature on Mpox infections. They may help inform public health interventions and clinical practice in managing and preventing Mpox outbreaks. Future studies should address these limitations and validate our findings in larger, more diverse populations and settings.

## Figures and Tables

**Table 1:** Demographic characteristics between Mpox-negative and -positive patients.

Characteristic	Overall, N = 38 ^1^	Mpox-Negative, n = 19 ^1^	Mpox-Positive, n = 19 ^1^	*p*-Value ^2^
Gender				0.008
Male	31 (82)	12 (63)	19 (100)	
Female	7 (18)	7 (37)	0 (0)	
Age (years)	34 (27, 40)	32 (16, 42)	35 (30, 40)	0.3
Gender identity				<0.001
Bisexual	3 (7.9)	0 (0)	3 (16)	
Homosexual	13 (34)	2 (11)	11 (58)	
Heterosexual	20 (53)	17 (89)	3 (16)	
MSM	2 (5.3)	0 (0)	2 (11)	
Transmission route				0.016
Health Services	1 (2.6)	1 (5.3)	0 (0)	
Non-Sexual contact	13 (34)	8 (42)	5 (26)	
Sexual contact	10 (26)	1 (5.3)	9 (47)	
Not specified	14 (37)	9 (47)	5 (26)	
Hospitalization	6 (16)	2 (11)	4 (21)	0.7
Other PCR Results				<0.001
VZV	3 (7.9)	3 (16)	0 (0)	
MPOX	19 (50)	0 (0)	19 (100)	
Unknown	16 (84)	16 (84)	0 (0)	

^1^ n (%); median (IQR). ^2^ Fisher’s exact test; Wilcoxon rank sum test.

**Table 2:** Clinical features between Mpox-negative and Mpox-positive patients.

Characteristic	Overall, N = 38 ^1^	Mpox-Negative, n = 19 ^1^	Mpox-Positive, n = 19 ^1^	*p*-Value ^2^
Clinical manifestations				
Fever	33 (87)	14 (74)	19 (100)	0.046
Headache	29 (76)	14 (74)	15 (79)	>0.9
Arthralgia	21 (55)	8 (42)	13 (68)	0.10
Nausea	7 (19)	2 (11)	5 (29)	0.2
Myalgias	26 (68)	11 (58)	15 (79)	0.2
Lumbago	15 (42)	5 (26)	10 (59)	0.048
Vomiting	1 (2.8)	0 (0)	1 (5.9)	0.5
Asthenia	22 (58)	7 (37)	15 (79)	0.009
Cough	10 (28)	4 (21)	6 (35)	0.3
Odynophagia	8 (22)	3 (16)	5 (29)	0.4
Chills	15 (41)	5 (26)	10 (56)	0.070
Diaphoresis	7 (19)	2 (11)	5 (29)	0.2
Number of symptoms	5.50 (3.00, 7.00)	4.00 (2.00, 6.00)	7.00 (3.00, 8.00)	0.015
Categorical number of symptoms				0.007
<7 symptoms	16 (52)	12 (75)	4 (27)	
>7 symptoms	15 (48)	4 (25)	11 (73)	
Bleeding ulcer	2 (5)	0 (0)	2 (12)	0.2
Painful ulcer	4 (11)	0 (0)	4 (24)	0.040
Comorbidities				
Diabetes	2 (5)	1 (5.3)	1 (5.3)	>0.9
None	6 (16)	2 (11)	4 (24)	0.4
Unknown comorbidities	30 (78)	16 (83.7)	14 (70.7)	
Cancer	1 (2.8)	0 (0)	1 (5.9)	0.5
Hepatitis C	0 (0)	0 (0)	0 (0)	
STI				
Gonorrhea	0 (0)	0 (0)	0 (0)	
Clamydia	0 (0)	0 (0)	0 (0)	
Syphilis	1 (2.6)	0 (0)	1 (5.3)	>0.9
Trichomoniasis	0 (0)	0 (0)	0 (0)	
PLWH	11 (29)	1 (5.3)	10 (53)	0.001
Pregnancy	0 (0)	0 (0)	0 (0)	

^1^ n (%); range, median (IQR). STI = sexually transmitted infections; PLWH = people living with HIV. ^2^ Pearson’s chi-squared test; Fisher’s exact test; Wilcoxon rank sum test.

**Table 3:** Characteristics and distribution of rash and lymphadenopathies.

Characteristic	Overall, N = 38 ^1^	Mpox-Negative, n = 19 ^1^	Mpox-Positive, n = 19 ^1^	*p*-Value ^2^
Skin lesions				
Macules	19 (53)	9 (47)	10 (59)	0.5
Papules	25 (69)	10 (53)	15 (88)	0.021
Vesicles	33 (87)	16 (84)	17 (89)	>0.9
Pustules	20 (56)	8 (42)	12 (71)	0.086
Scabs	22 (61)	9 (47)	13 (76)	0.074
Affected regions				
Head	22 (58)	10 (53)	12 (63)	0.5
Face	26 (68)	12 (63)	14 (74)	0.5
Neck	20 (53)	11 (58)	9 (47)	0.5
Chest	23 (64)	10 (53)	13 (76)	0.14
Upper limbs	30 (81)	17 (89)	13 (72)	0.2
Lower limbs	25 (68)	13 (68)	12 (67)	>0.9
Mouth	3 (8.3)	2 (11)	1 (5.9)	>0.9
Genital area	13 (35)	5 (26)	8 (44)	0.2
Abdomen	13 (36)	7 (37)	6 (35)	>0.9
Back	18 (50)	11 (58)	7 (41)	0.3
Perianal area	4 (11)	2 (11)	2 (12)	>0.9
Soles of feet	6 (17)	4 (21)	2 (12)	0.7
Palms	13 (36)	6 (32)	7 (41)	0.5
Affected regions				0.069
Cephalocaudal	18 (50)	12 (63)	6 (35)	
Centrifuge	9 (25)	6 (32)	3 (18)	
Centripetal	2 (5.6)	0 (0)	2 (12)	
Simultaneous	3 (8.3)	1 (5.3)	2 (12)	
Other	1 (2.8)	0 (0)	1 (5.9)	
Not specified	3 (8.3)	0 (0)	3 (18)	
Lymphadenopathies, affected regions:				
Axylar	4 (11)	0 (0)	4(24)	0.040
Cervical	12 (32)	4 (21)	8 (42)	0.2
Inguinal	6 (17)	1 (5.3)	5 (29)	0.081
Other	6 (17)	1 (5.3)	5 (29)	0.081
Other location:				>0.9
Retroauricular	1 (17)	0 (0)	1 (20)	
Submandibular	5 (83)	1 (100)	4 (80)	

^1^ n (%). ^2^ Pearson’s chi-squared test; Fisher’s exact test.

**Table 4:** Univariate and multivariate logistic regression analysis of covariates to predict Mpox-positive patients.

Characteristic	Univariate Model			Multivariate Model 1			Multivariate Model 2		
	OR ^1^	95% CI ^1^	*p*-Value	OR ^1^	95% CI ^1^	*p*-Value	OR ^1^	95% CI ^1^	*p*-Value
Lumbago	4	1.02, 17.6	0.053	0.48	0.02, 10.6	0.6			
Asthenia	6.43	1.62, 30.3	0.012	0.71	0.02, 24.7	0.8			
Papules	6.75	1.38, 50.8	0.03	8.12	0.30, 2.441	0.3			
Number of symptoms	1.4	1.08, 1.91	0.017	0.46	0.11, 1.28	0.2			
Number of symptoms									
<7 symptoms	—	—		—	—		—	—	
>7 symptoms	8.25	1.79, 47.0	0.01	670	2.36, 7.394	0.068	10.3	1.47, 119	0.031
Number of lymphadenopathies by regions									
<1 affected region	—	—		—	—		—	—	
>1 affected region	9.1	2.16, 46.7	0.004	62.8	2.03, 15.431	0.051	8.67	1.2, 97.8	0.044
PLWH	20	3.12, 398	0.008	14.3	1.08, 505	0.073	16	1.39, 53	0.054

^1^ OR = odds ratio; CI = confidence interval; PLWH = people living with HIV.

## Data Availability

The datasets obtained and analyzed during the research are accessible upon reasonable request from the corresponding authors.
